# Inter and intra-annual links between climate, tree growth and NDVI: improving the resolution of drought proxies in conifer forests

**DOI:** 10.1007/s00484-021-02170-5

**Published:** 2021-07-15

**Authors:** Marín Pompa-García, J. Julio Camarero, Michele Colangelo, Marcos González-Cásares

**Affiliations:** 1 Facultad de Ciencias Forestales de la, Universidad Juárez del Estado de Durango, Río Papaloapan Y Blvd., Durango S/N Col. Valle del Sur, Durango, México; 2 Instituto Pirenaico de Ecología (IPE-CSIC), Avda. Montañana 1005, Apdo. 202, 50192 Zaragoza, Spain; 3 School of Agricultural, Forest, Food and Environmental Sciences, Univ. Basilicata, Potenza, Italy

**Keywords:** Xylogenesis, Earlywood, Latewood, Drought, *Pinus teocote*, Dendroecology

## Abstract

The inter- and intra-annual variability in radial growth reflects responses to climatic variability and water shortage, especially in areas subjected to seasonal drought. However, it is unknown how this variability is related to forest productivity, which can be assessed by measuring changes in canopy greenness and cover through remote sensing products as the Normalized Difference Vegetation Index (NDVI). We combine xylogenesis with measurements of inter-annual changes in seasonal wood production (earlywood width, adjusted latewood width) and NDVI to improve the understanding of climate and drought impacts on growth and forest productivity in a *Pinus teocote* stand located in northern Mexico. Cambial dynamics accelerated in March and a high production of radially enlarging and thickening tracheids were observed from April to October and from June to October, respectively. Tracheid maturation was very active in October when latewood production peaked. Wet conditions in winter-spring and summer-autumn enhanced earlywood and latewood production, respectively. Earlywood and latewood were constrained by long (4–10 months) and short (2–3 months) droughts, respectively. The earlywood production depended on April soil moisture, which agrees with the peak of radially enlarging tracheid production found during that month. Aligning drought proxies at inter- and intra-annual scales by using growth and productivity measures improves our understanding of conifer forest responses to water shortage.

## Introduction

The world’s forests are the largest carbon reservoir in terrestrial ecosystems, which affect global carbon fluxes and climate change mitigation (Pan et al. 2011). Within trees, woody tissues account for 15% of anthropogenic carbon dioxide emissions every year (Cuny et al. 2015). Therefore, understanding processes of xylem formation (xylogenesis) and radial growth at inter- and intra-annual scales is mandatory for quantifying carbon fixation and evaluating climate mitigation of forests (Vaganov et al. 2006).

The carbon sink of forests subjected to seasonal drought depends on annual and seasonal variability in precipitation showing the outstanding role of soil moisture as driver of radial growth and carbon uptake (Humphrey et al. 2018). To complement the understanding of the dynamics of radial growth in response to drought, a better temporal resolution of tree-ring proxies is required (Timofeeva et al. 2017). In conifers, the measurement of earlywood and latewood widths is often used to study seasonal growth responses to climate and drought (e.g., Griffin et al. 2011, 2013; Cabral-Alemán et al. 2017; Camarero et al. 2020).

The earlywood formation depends on carbohydrates synthesized during the prior year, whereas latewood depends on current-year photosynthates (Kagawa et al. 2006). However, earlywood and latewood widths are very related, which requires removing part of this non-biological relationship to isolate relevant climatic and drought signals of latewood related to late-season growing conditions. For instance, a high summer-autumn moisture improves latewood production in seasonally dry regions (Meko and Baisan 2001). The resulting variable, after removing part of the earlywood − latewood association, is referred to as adjusted latewood, and contains climatic information different than earlywood and latewood (Griffin et al. 2011; Torbenson et al. 2016; Collado et al. 2018). The drought impacts on earlywood and adjusted latewood should be interpreted on the basis of xylogenesis, which allows reconstructing seasonal cambial dynamics and how they are coupled to climate (Camarero et al. 2010).

As a further effort to improve the explanations of climate-growth relationships at annual and seasonal scales, remote sensing data have been used to monitor changes in forest productivity and canopy greenness (e.g., Pompa-García et al. 2021). As a result, remote sensors are a flexible, reliable and a widely recognized tool to study the intra-annual changes in radial growth and canopy greenness in forests (Decuyper et al. 2020). Among the most used remote sensing variables, the Normalized Difference Vegetation Index (NDVI) is considered as a valuable proxy to investigate the effects of climate variability on vegetation phenology and productivity due to its close relationship with plant activity and canopy greenness and cover (Gazol et al. 2018; Pompa-García et al. 2021). Thus, when radial-increment dynamics data and remote sensing are integrated, the explanatory capacity of these variables allows disentangling ecological strategies used by tree species to withstand drought stress (Vicente-Serrano et al. 2020).

Northern Mexico is a very diverse and drought-prone region where the integration of tree-ring and remote-sensing proxies is critical to assess forest responses to seasonal water shortage (González-Casares et al. 2017, 2020). Linking inter- and intra-annual variability with remote sensing data could provide finer resolution proxies for growth dynamics associated with climate variability that are still at an early stage. There are some studies dealing with the climatic drivers of tree growth and NDVI data in high-elevation forests from central Mexico (Correa-Díaz et al. 2019, 2020), and in semi-arid forests from northern Mexico and the south-western USA (Villanueva-Díaz et al. 2009; Pompa-García et al. 2013; González-Casares et al. 2017, 2020; Szejner et al. 2020). However, intra-annual xylogenesis studies are still scarce in this region (but see Belmecheri et al. 2018; Ziaco et al. 2018; Pompa-García et al. 2021). Therefore, here we aim to analyze the inter- and intra-annual links between climate, tree growth and NDVI data of *Pinus teocote*, a conifer species abundant in the study region. We hypothesize that seasonal radial growth, in particular LW formation, would be a more robust proxy of intra-annual changes in water shortage as compared with NDVI since the former is a low-priority carbon sink. We argue that the integration of these variables improves the temporal resolution and links between forest drought proxies, and it is of strategic ecological value in the face of climate warming and forecasted aridification.

## Materials and methods

### Study area and tree species

**Fig. 1:**
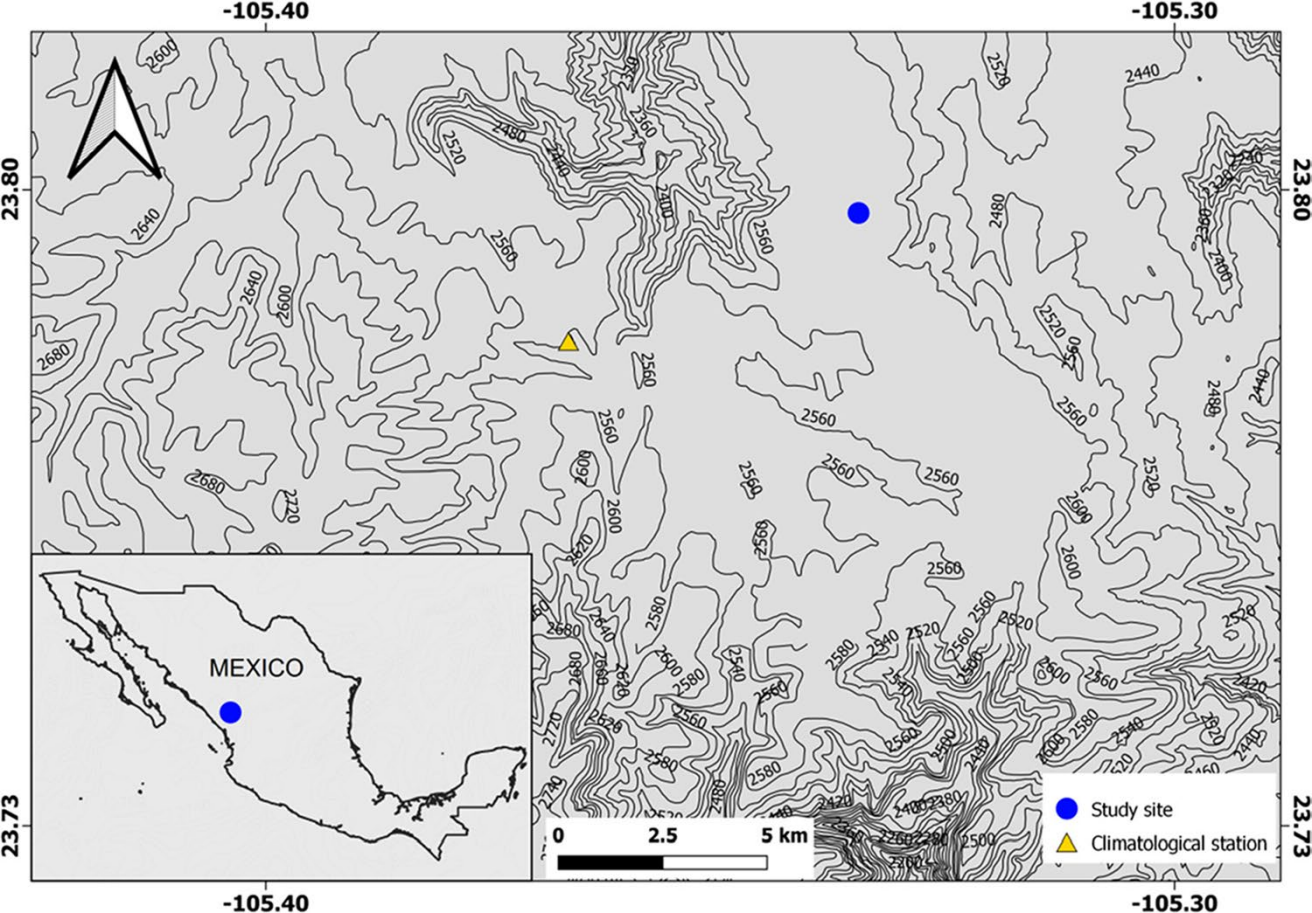
Map of the study area in north-western Mexico showing the location of the study site and the climatological station (El Salto)

The study area is located in El Salto (Pueblo Nuevo, Durango, north-western Mexico), in the Sierra Madre Occidental, at 23.4° N, 105.2° W and an elevation of 2598 m a.s.l (Fig. 1). This region has a temperate, sub-humid climate with a wet cool summer because of the North American Monsoon influence (Seager et al. 2009; Torbenson et al. 2016). The study site presents a mean annual temperature of 11.5 °C, and total annual precipitation of 1200 mm (CNA 2020). The rainy season starts in late June to early July, representing 35% of annual precipitation. A second wet season occurs from November to February, representing 14% of annual precipitation. The vegetation is dominated by several pine species such as *Pinus engelmannii* Carr. var. *blancoi* (Martínez) and *Pinus lumholtzii* B.L. Rob. and Fernald, and oak species such as *Quercus durifolia* Seemen, *Quercus rugosa* Née, and *Quercus sideroxyla* Bonpl. (Villaseñor 2016). The soils correspond to the luvisol type, with high clay content (González-Cásares et al. 2020). The selected study site is homogeneous in terms of local climatic conditions, vegetation composition and soil type.

The studied species (*Pinus teocote* Schiede ex Schltdl. and Cham) is distributed from the southern USA to southern Mexico, being one of the most widespread pine species in Central America. Trees of 20–25 m in height and mean diameter at 1.3 m up to 75 cm can be found, mainly in open forests and associated with oaks and other pine species at altitudes between 1100 and 3300 m, and occupying varied habitats but mainly on well-drained, thin calcareous soils (Farjon 2010). Trees of this pine species are found across the study region in sites with 500 − 1000 mm annual precipitation, and where frosts are rare.

### Climate data

We obtained local climate data from the nearby “El Salto” (23° 47’ N, 105° 22’ W, 2560 m a.s.l) station (CNA 2020). First, we obtained monthly and seasonal values of mean maximum (Tmax) and minimum (Tmin) temperatures and precipitation for the period 1968–2019 (Fig. S1). Second, we obtained daily climate data for the years 2018 and 2019 when the intra-annual xylogenesis sampling was done.

The Standardized Precipitation-Evapotranspiration Index (SPEI) drought index was used to quantify drought severity and duration (Vicente-Serrano et al. 2010). The SPEI is a multi-scalar index that quantifies drought intensity according to the difference between the atmospheric evaporative demand and precipitation and considering different periods of cumulative climatic water balance. Negative values of the SPEI indicate water scarcity with values below − 1.5 usually indicating drought. We calculated SPEI data from the local climate series using the SPEI R library (Beguería et al. 2014).

Finally, since soil moisture plays a key role in forest functioning (Pan et al. 2019), we obtained the 10-cm soil moisture data at ca. 1° spatial resolution for the period 1979–2016 from the ERA-Interim global atmospheric reanalysis (Dee et al. 2011; Dorigo et al. 2017).

### Dendrochronological methods

Sixteen dominant trees of *P. teocote*, the most abundant tree species in the study stand (basal area > 70%), were sampled. Two radial increment cores per individual were obtained at breast height (1.3 m) using a Pressler borer. Cores were mounted on woody grooves and polished with progressively finer sandpaper until rings were clearly visible. Visual cross-dating of the series was performed and measurement of ring width, earlywood and latewood widths was made to a resolution of 0.01 mm using a Velmex measuring system. Visual dating was statistically validated using the COFECHA program (Holmes 1983). To exclude biological and geometric trends unrelated to climate, ring, earlywood and latewood width series were detrended and pre-whitened by fitting negative exponential functions and autoregressive models, respectively (Fritts 1976). Residual mean series or chronologies of ring, earlywood and latewood series were obtained using bi-weight robust means. Standardization and construction of chronologies were carried out using the dplR (Bunn 2010) of the R software (R Development Core Team, 2020).

The resulting earlywood and latewood width chronologies were characterized by calculating several statistics: the first-order autocorrelation (AC) which quantifies the year-to-year persistence in width; the mean sensitivity (MS), a measure of the relative change in ring-width index between consecutive years; and the mean correlation between series (rbar) (Fritts 1976). We also obtained the Expressed Population Signal (EPS) to determine how coherent and replicated the earlywood and latewood chronologies were, with a minimum threshold value of 0.85 being usually considered to define well-replicated chronologies (Wigley et al. 1984).

To remove the influence of earlywood variability on latewood series, we followed Meko and Baisan (2001), and calculated adjusted latewood by obtaining the residuals of a earlywood − latewood linear regression using the chronologies of both variables. We used the following linear regression to calculate adjusted latewood (LWadj):

1 $$\mathrm{L}\mathrm{W}\mathrm{a}\mathrm{d}\mathrm{j}=\mathrm{L}\mathrm{W}-\left(0.6246\mathrm{E}\mathrm{W}+0.39\right)$$

with a fit of R^2^ = 0.78 (*p* < 0.01). The aim was to isolate the influence of summer-autumn climate conditions on the seasonal wood formation during the late growing season. Previous studies reported that adjusted latewood contained different information from that recorded by earlywood (EW) and latewood (LW) (e.g., Torbenson et al. 2016; Collado et al. 2018).

### Xylogenesis assessment

Wood microcores (15 mm long, 2 mm diameter) were extracted biweekly from April 16 2018 through April 13 2019, using a Trephor® microborer (Rossi et al. 2006). Samples were obtained approximately 5 cm apart following a spiral on the stem at a height of 1.0–1.5 m from seven trees and stored in a 50% ethanol solution. We selected dominant trees with a diameter (mean ± 1SE) at 1.3 m of 28.0 ± 2.4 cm, a total height of 9.6 ± 0.4 m and age (estimated by taking basal cores) of 67 ± 4 years.

In the laboratory, each microcore was vertically oriented following the fibers direction. Then each sample was cut with a 15–20 μm thickness using a sliding microtome (Leica SM 2010 R) with temperature Controlled Freezing Stages for Microtomes (Physitemp BFS-30MP), which allowed slowly freezing the wood samples for optimal sectioning. The sections were mounted on glass slides, stained with 0.05% cresyl violet acetate (Antonova and Shebeko 1981), and fixed using Eukitt®. Then, sections were observed under bright field and polarized light after coloration within 10–30 min of staining at 100–500 magnification (Rossi et al. 2006). Images were photographed with a digital camera (JVC 3.3 CCD GC-X3E; JVC, Yokohama, Japan) to verify cell counts and to distinguish earlywood and latewood tracheids following Denne (1988). EW and LW tracheids were characterized by presenting wide or narrow radial lumen and thin or thick walls, respectively.

**Fig. 2:**
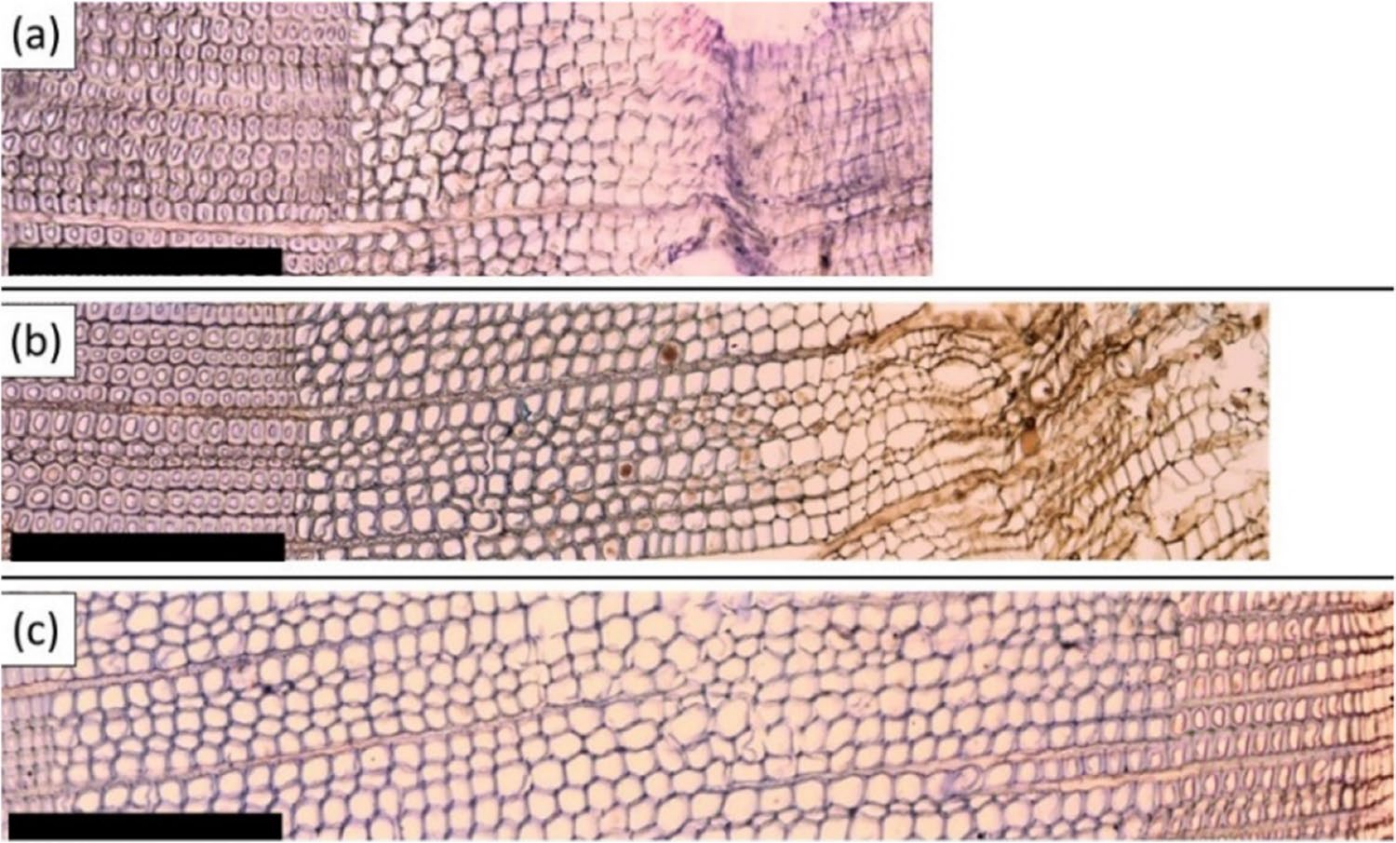
Images showing the major phases of xylogenesis in *Pinus teocote* during 2018: (**a**) radial enlargement of tracheids (late April), (**b**) wall thickening (early June), and (**c**) tracheid maturation (early December). The scale bars measure 0.1 mm. The bark is situated to the right of the images

For each sample, we counted and calculated averages along five radial files of the number of: cells in the cambial zone, radially enlarging tracheids, cell-wall thickening tracheids, and earlywood and latewood mature tracheids. According to developmental stage (Antonova and Stasova 1993), cells stained a different color and presented different shapes (Fig. 2). Cambium cells have similar and small radial diameters with thin primary cell walls. Radially enlarging tracheids presented protoplast enclosed by a thin wall but showed radial increment. The end of radial-cell expansion and the onset of secondary cell wall formation corresponded to the appearance of cell corner rounding and lignification in thickening tracheids showing blue-stained walls. Lastly, tracheid maturation ended when traces of cytoplasm were not detected in the lumen and walls were stained blue.

The numbers of cells in the five radial files per tree were averaged to assess the onset, the period of maximum production of mature cells and the end of xylem formation. The onset of xylem formation was regarded as the date (DOY, day of year) when at least five trees showed more than one row of cells in the cell-enlargement phase (Camarero et al. 2010). The ending of xylogenesis was considered the date when all trees lacked tracheids corresponding to the cell-wall thickening phase. We calculated the rate of production of earlywood and latewood mature tracheids by subtracting successive values of summed tracheids and dividing them by the elapsed time between samplings (Camarero et al. 1998).

### NDVI data

Using the Google Earth Engine, as a geospatial processing platform cloud-based for large-scale, multi-temporal environmental monitoring and analysis (Gorelick et al. 2017), we calculated NDVI = (NIR − R / (NIR + R) for the period 1999–2017, where NIR and R are near infrared and red values, respectively. Google Earth Engine provides access to petabytes of satellite imagery and machine learning algorithms using Google computational infrastructure (Gorelick et al. 2017) through the arrangement of MODIS (Moderate-Resolution Imaging Spectroradiometer) images (Didan 2015).

### Statistical analyses

To analyze the intra-annual scales of growth (xylogenesis), we converted daily climate data (Tmax, Tmin, precipitation) into 5-, 10-, and 15-day averages (temperatures) or sums (precipitation) to capture differently resolved or lagged climatic influences on cell production (see Gutiérrez et al. 2011). Then, Spearman correlations (*r*_*s*_) were calculated between the main xylogenesis phases (numbers of cambium cells, number of enlarging, thickening and mature tracheids) and the climate data calculated at several temporal resolutions.

To evaluate the impact of inter-annual climate variability on earlywood and adjusted latewood, Pearson correlation coefficients (*r*) were calculated between residual chronologies and monthly climatic variables (Tmax, Tmin and precipitation). Associations between earlywood and adjusted latewood with the SPEI drought index were evaluated at 1- to 30-month long scales also using Pearson correlations. To quantify associations between seasonal growth and NDVI, Pearson correlations were calculated between earlywood, adjusted latewood and monthly NDVI values. All analyses were carried out using the R statistical package (R Development Core Team, 2020).

## Results

### Xylogenesis

**Fig. 3:**
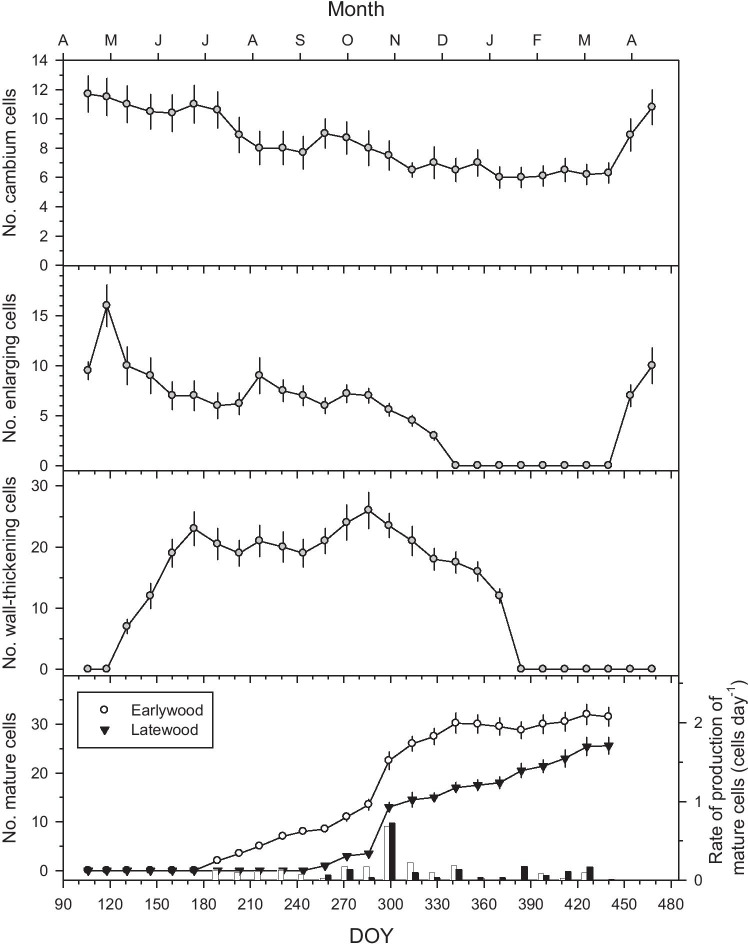
Number of xylem cells according to their development stage observed in *Pinus teocote*. In the lowermost graph, the numbers of mature earlywood and latewood tracheids are presented, and empty and filled bars correspond to the rates of production (right y axis) of earlywood and latewood mature cells, respectively. The lowermost x axis shows the day of the year (DOY) from 16 April 2018 to 13 April 2019 and the uppermost x axis shows months. Values are means ± SE

The number of cambium cells peaked from April to May (DOY 103–131), whereas the number of radially enlarging tracheids peaked in late April (DOY 118) and showed minimum values from December to February suggesting cambial onset occurs in March (DOY 89) (Figs. 2 and 3). The number of thickening tracheids peaked from June to December (DOY 160–356), whereas the rates of production of earlywood and latewood mature tracheids peaked from mid to late October (DOY 286–299) increasing afterwards and reaching maximum values in winter. Radially-enlarging tracheids reached a maximum in late April (DOY 188) followed by a secondary peak of production in early August (DOY 216). The production of thickening tracheids reached two maxima in late June (DOY 174) and mid-October (DOY 286).

Correlation analyses between daily climate data and xylogenesis phases peaked for 10-day periods and showed a positive association between maximum temperature and the number of radially-enlarging tracheids (*r*_*s*_ = 0.46; *p* = 0.04). The number of thickening tracheids was significantly correlated to precipitation (*r*_s_ = 0. 47; *p* = 0.03).

### Earlywood and latewood width chronologies and links with NDVI

Earlywood and latewood width chronologies showed similar first-order autocorrelation, but the MS, rbar and EPS of earlywood series were higher than in the case of latewood series (Table 1). The EPS was above the 0.85 threshold in both cases.

**Table 1:** Dendrochronological statistics of earlywood and latewood width chronologies of *Pinus teocote* considering the best-replicated period 1968–2017

Variable	Mean ± SD (mm)	AC	MS	rbar	EPS
Earlywood	1.54 ± 0.839	0.48	0.43	0.61	0.95
Latewood	0.54 ± 0.271	0.42	0.37	0.47	0.89

*SD*, Standard deviation; *AC*, first-order autocorrelation; *MS*, Mean sensitivity; *rbar*, mean correlation between series; *EPS*, Expressed population signal.

**Fig. 4:**
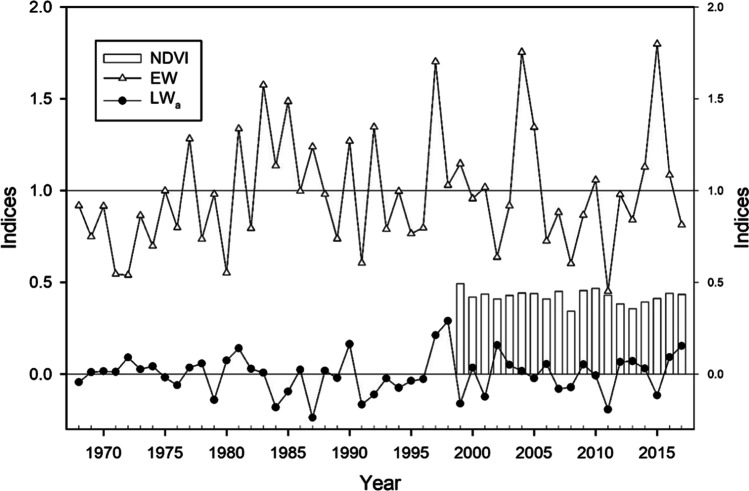
Chronologies of earlywood (EW) and adjusted latewood (LW_a_) width of *Pinus teocote* considering the best replicated period 1968–2017. Bars represent annual NDVI for the period 1999–2017 (right y axis)

The year-to-year variability of earlywood was elevated with the following years of high and low earlywood width indices: 1998, 2005, 2015 and 1980, 2002, 2011, respectively (Fig. 4). In the case of adjusted latewood, the largest increases occurred in 1990, 1998, and 2002, while the lowest values were recorded in 1987, 2002, and 2011. We did not find any significant correlation between earlywood and NDVI or between adjusted latewood and NDVI.

### Climate- and drought-growth/NDVI relationships

**Fig. 5:**
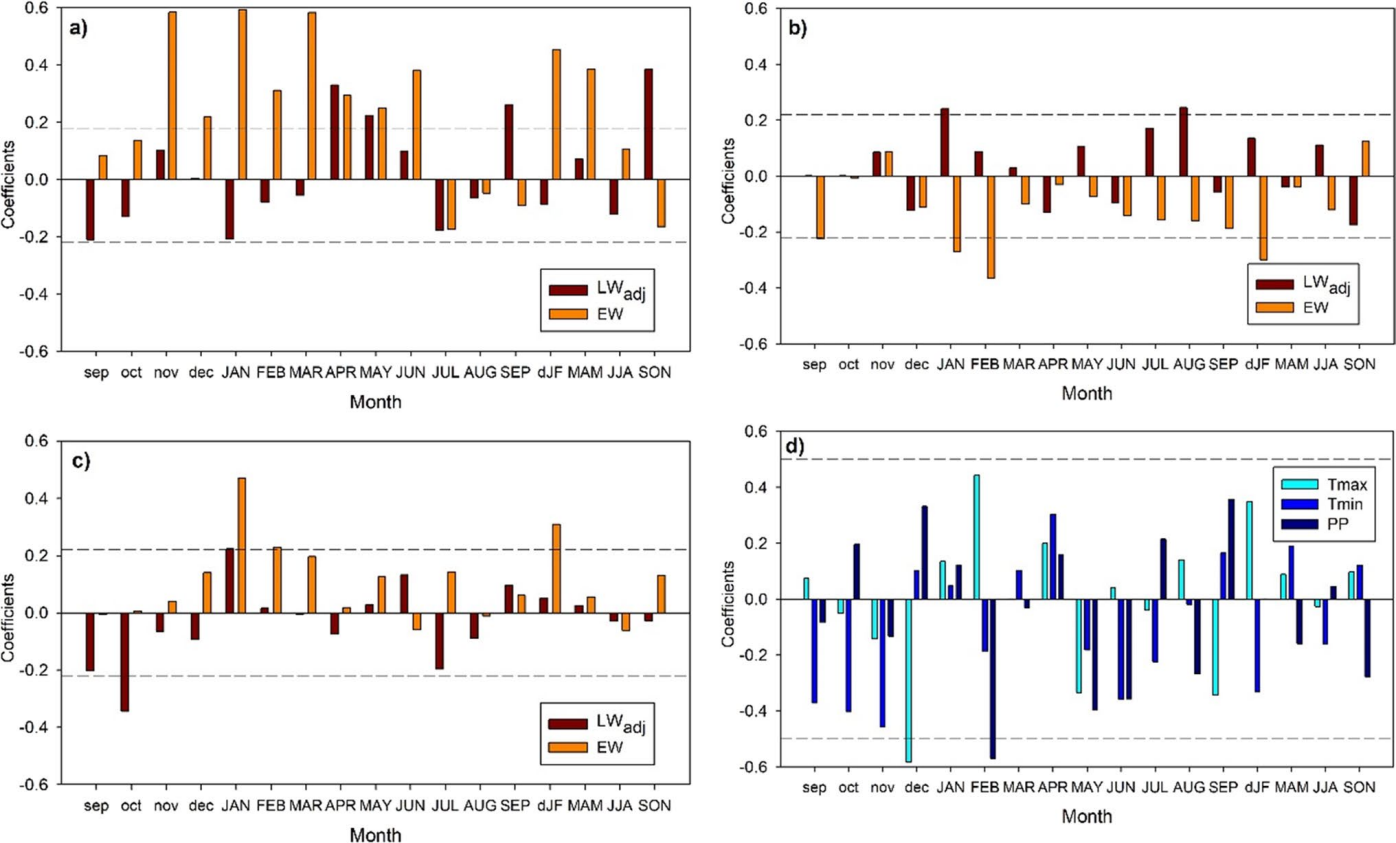
Climate- and NDVI-growth associations. Pearson correlation coefficients were calculated by relating earlywood (EW) and adjusted latewood (LWadj) indexed series with precipitation (**a**), mean maximum temperature (**b**) and mean minimum temperature (**c**). The NDVI data were also related to these climatic variables (Tmax, mean maximum temperature; Tmin, mean minimum temperature; PP, precipitation) (**d**). The horizontal dashed lines indicate the 0.05 significance levels. The prior and current year months are abbreviated by lowercase and uppercase letters, respectively. Seasons are abbreviated as follows: winter, dJF; spring, MAM; summer, JJA; and autumn, SON

The earlywood was positively and significantly influenced by the precipitation during the previous November and January to March of the growing year (Fig. 5a), and also by prior winter and current spring precipitation. Elevated maximum temperatures in the previous September and winter, and in the current January and February were negatively related to earlywood (Fig. 5b), but high minimum January and winter temperatures were positive related to earlywood (Fig. 5c). On the other hand, adjusted latewood positively correlated with April, September and autumn precipitation (Fig. 5a), and was also positively associated with maximum temperatures of January and August (Fig. 5b). In contrast, adjusted latewood was negatively influenced by elevated minimum temperatures of the previous October (Fig. 5c).

Regarding NDVI-climate associations, the NDVI increased in response to low prior December maximum temperatures and low precipitation in the current February (Fig. 5d). A high soil moisture in the prior October was positively related to NDVI (*r* = 0.53, p = 0.025).

**Fig. 6:**
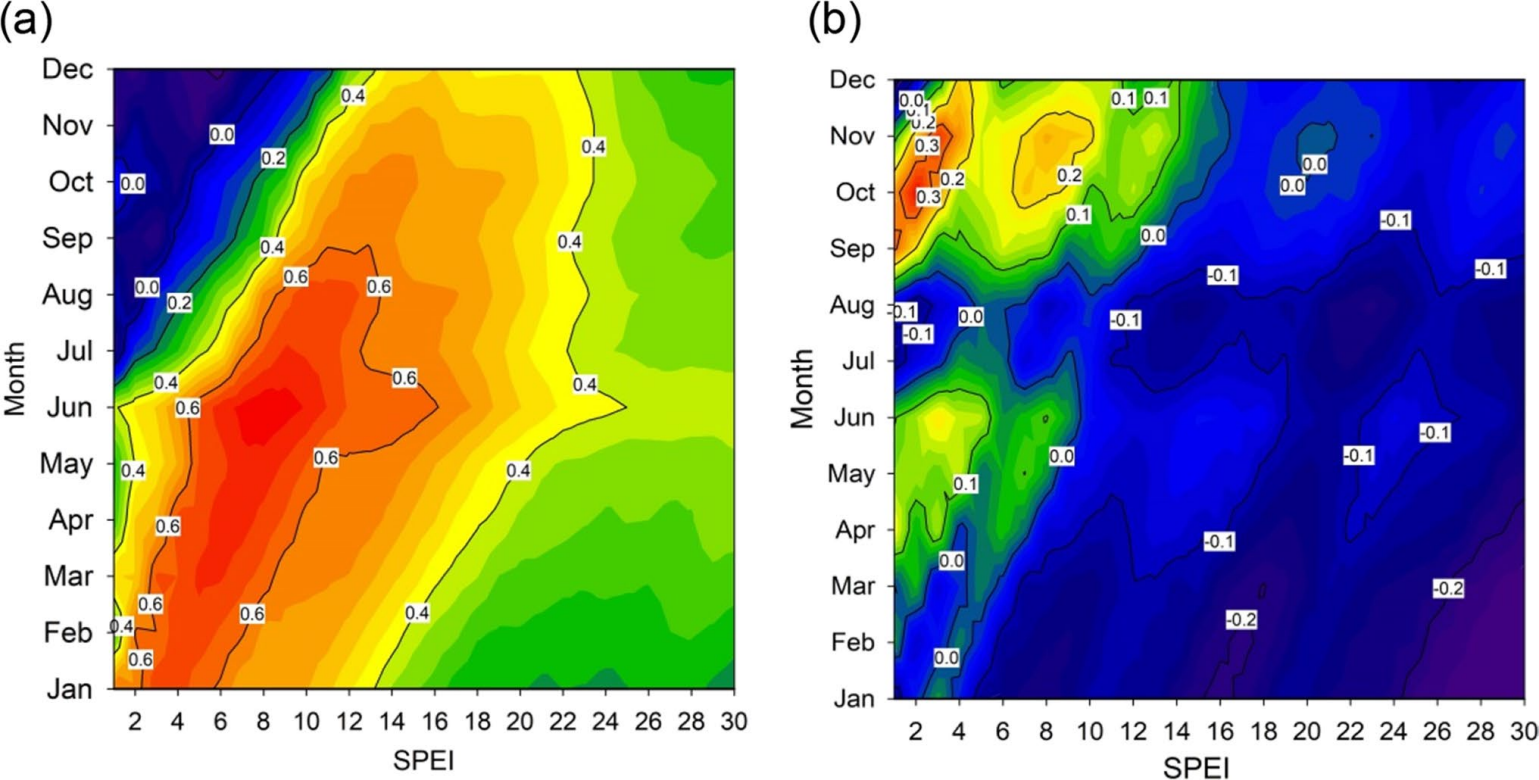
Growth-SPEI associations. Pearson correlation coefficients calculated between the SPEI drought index and earlywood (**a**) and adjusted latewood (**b**) chronologies. Correlation values are significant (p < 0.05) at *r* > 0.22

The earlywood showed responses to 4- to 10-month SPEI values from April to July (Fig. 6a), while adjusted latewood responded to 2- to 4-month SPEI values from October to November (Fig. 6b), when latewood production peaked (Fig. 3).

**Fig. 7:**
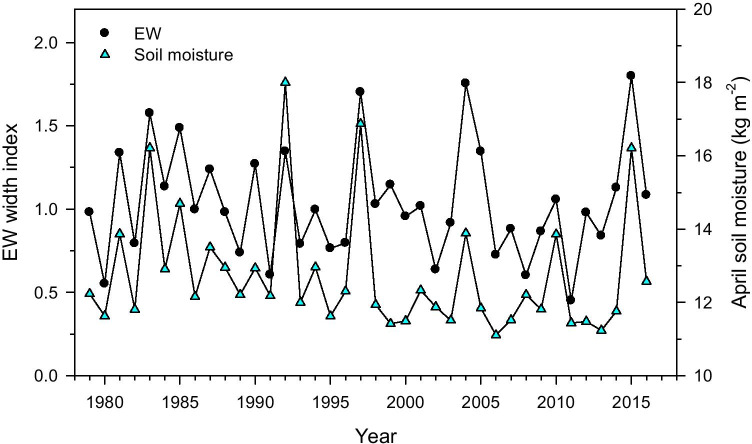
Common variability between the earlywood (EW) chronology and April soil moisture

Finally, we found a strong positive association (*r* = 0.75, *p* < 0.0001) between earlywood growth rates and April soil moisture (Fig. 7).

## Discussion

### NDVI and tree growth responses to climate

We analyzed the inter- and intra-annual links between climate, xylogenesis, seasonal radial growth and NDVI in *Pinus teocote,* a pine species inhabiting a seasonal dry region. Despite that we hypothesized latewood to be a more robust proxy of intra-annual changes in water shortage as compared with NDVI, we found that earlywood showed the strongest responses to changes in precipitation and estimated soil moisture. The strong response of earlywood to April soil moisture (Fig. 7) is supported by the xylogenesis data since April is the month when the number of radially enlarging tracheids peaked (Fig. 3), and radially enlarging tracheids are tightly related to annual growth rates (Horáček et al. 1999). As tested in early studies (Ziaco et al. 2018; Pompa-García et al. 2021), the availability of water before and during early spring, when temperatures start rising, seems to explain the rate of cellular production. Also wood anatomical features mirror the role of climatic conditions by increasing the number of radially-enlarging tracheids (Fig. 3), which have implications on the coming xylogenesis phases (Cuny et al. 2015).

We also detected a positive response of NDVI to April precipitation (Fig. 5d), suggesting an increase in greenness of *P. teocote* canopy during years with wet spring conditions. Our results agree with previous dendrochronological studies in other conifers from drought-prone northern Mexico (Pompa-García et al. 2017; Acosta-Hernández et al. 2020). Climate conditions during the previous winter modulate the extension of radial growth on spring because wet-cool winter conditions recharge soil moisture and improve growth and cell expansion which translates into the production of more and wider tracheids (Cabral-Alemán et al. 2017; Acosta-Hernández et al. 2020; Pasho et al. 2012). In September, both LWadj and NDVI showed positive responses to high precipitation (Fig. 5d), and during that month tracheid wall-thickening and maturation increased (Fig. 3). This would explain why adjusted latewood showed stronger responses to mid and long droughts during the mid to late growing season, whereas earlywood was more sensitive to short droughts in the early growing season (Fig. 6). These results are supported by studies on carbon partitioning since latewood formation involves active cell-wall maturation and lignification, two processes which require large amounts of carbohydrates (Kagawa et al. 2006).

Warm conditions during the previous winter exacerbate atmospheric moisture demand leading to water shortage (higher vapor pressure deficit) and decreased earlywood production (Fig. 5b), which could lead reduce forest productivity as indicated the negative association between NDVI and prior December maximum temperature (Fig. 5d). This result evidences the dependency of earlywood production on carbohydrates produced during the prior year and could explain carryover effects such as drought legacies, i.e. lasting impacts of drought on tree growth and productivity (Anderegg et al. 2015; Szejner et al. 2020).

### Climatic drivers of seasonal wood production

Soil moisture variability explains better radial growth rates than precipitation as already observed in Mediterranean pine forests (Sarris et al. 2013; Camarero et al. 2021). We observed a high covariance between April soil moisture and earlywood (Fig. 7), confirming that soil moisture availability is an ultimate factor of growth and wood production. Therefore, we can speculate that the availability of soil water pools stored during the previous winter determines spring growth (earlywood production) and the final growth rate. Thus, drying up of deeper soil layers in winter-spring may lead to lagged growth reductions, drought legacies and long-term declines in forest productivity (Anderegg et al. 2015). Species-specific analysis demands more research on how the access and use of soil water stored at different depths impact on tree radial growth and forest productivity by using water H and O isotopes. A caveat concerning our analyses is the use of reanalysis data for soil moisture, so actual measurements should be taken and used in future studies and considering several sites. Alternatively, reanalysis data should be compared and corrected with field soil moisture measurements.

The summer Monsoon regime dominant in the study area (Seager et al. 2009) determines the responses of adjusted latewood to summer precipitation as already shown by Torbenson et al. (2016). Earlywood and adjusted latewood chronologies have potential to explain seasonal climate variation in the study area (e.g., Figs. 5, 6 and 7), which was not explained by ring width. The adjusted latewood series seem to record autumn precipitation signals due to an extension of the growing season, potentially related to tracheid wall thickening and production of latewood cells in the late growing season (Fig. 3), and linked to a premature onset of Monsoon rains in the area (Pompa-García et al. 2017). Thus, the soil moisture source is available for a longer period of time which may increase hydraulic conductivity and photosynthesis rate, augment the rate of formation of tracheids and increase wood production (Sarris et al. 2013). It should be further investigated how the shift in the Monsoon season affects the shift from earlywood to latewood formation to better understand changes in tree growth and water use. Overall, the adjusted latewood is a valuable dendroecological proxy recording a distinct climatic signal linked to summer-autumn rainfall and the Monsoon regime (see Griffin et al. 2011, 2013), although further research efforts on tree-age effects on adjusted latewood are encouraged.

### Linkages between NDVI, tree growth and xylogenesis

We noted the existence of lagged responses of tree growth to climate evidenced from the xylogenesis and NDVI variability in *P. teocote* which agree with Belmecheri et al. (2018), and could be explained by high photosynthesis rates during sunny-warm days with wet soils in late winter and early spring. Xylogenesis keeps more synchrony with changes in soil moisture. Therefore, a better quantification of the timing of wood formation and NDVI is needed in other tree species. For instance, modeling NDVI from xylem duration and length of phenology (such as budburst, shoot and needle growth) could refine mechanistic approaches towards xylem formation. Our approach assumed that NDVI values at the pixel level are representing the trees sampled; however, our spatial data considered coexisting tree and shrub species and the understory, which have wider seasonal dynamics. On the contrary, the xylogenesis was studied at individual level and at finer temporal scale. We argue that unmanned aerial vehicles may solve some inconsistencies between different spatial scales and provide solutions to ecological questions at finer spatial and temporal resolutions (Gallardo-Salazar et al. 2021).

Interestingly, our climate-growth relationships were supported by the analyses of seasonal wood production. This may mean that the summer Monsoon will gain importance in the face of forecasted drought scenarios for North American semi-arid conifer forests (Cook and Seager 2013). If projected increases in aridification continue and lead to drier soils in winter and spring, we would expect a decrease in earlywood production accompanied by more frequent long-term growth declines, widespread dieback episodes and loss in forest productivity. Thus, our quantitative approach is a clear evidence of the complexity of hydroclimatic drivers than contribute to wood formation at intra-annual scales. Nevertheless, we improved our understanding of cambial phenology, wood formation and forest productivity in a drought-probe conifer by combining several proxies (e.g. NDVI, earlywood, adjusted latewood, xylogenesis).

## Conclusions

We demonstrated that studying growth at inter- and intra-annual scales in *Pinus teocote* enhanced our understanding of drought constraints on forest productivity. In particular, analyzing xylogenesis and considering earlywood and adjusted latewood allowed detecting the impact of spring soil moisture on earlywood, cambial dynamics and NDVI. Further approaches should focus on earlywood and latewood chronologies and relate them to seasonal climatic drivers in widely distributed conifers (e.g., *Pseudotsuga menziesii*, *Pinus ponderosa*, *Pinus sylvestris*) across different dry regions of Northern America and Eurasia.
